# Validity of six consumer-level activity monitors for measuring steps in patients with chronic heart failure

**DOI:** 10.1371/journal.pone.0222569

**Published:** 2019-09-13

**Authors:** Tomas Vetrovsky, Michal Siranec, Jitka Marencakova, James J. Tufano, Vaclav Capek, Vaclav Bunc, Jan Belohlavek

**Affiliations:** 1 Faculty of Physical Education and Sport, Charles University, Prague, Czech Republic; 2 2nd Department of Medicine – Department of Cardiovascular Medicine, First Faculty of Medicine, Charles University and General University Hospital in Prague, Prague, Czech Republic; 3 Second Faculty of Medicine, Charles University, Prague, Czech Republic; UNSW Sydney, AUSTRALIA

## Abstract

**Introduction:**

Although numerous activity trackers have been validated in healthy populations, validation is lacking in chronic heart failure patients who normally walk at a slower pace, making it difficult for researchers and clinicians to implement activity monitors during physical activity interventions.

**Methods:**

Six consumer-level activity monitors were validated in a 3-day field study in patients with chronic heart failure and healthy individuals under free living conditions. Furthermore, the same devices were evaluated in a lab-based study during treadmill walking at speeds of 2.4, 3.0, 3.6, and 4.2 km·h^-1^. Concordance correlation coefficients (CCC) were used to evaluate the agreement between the activity monitors and the criterion, and mean absolute percentage errors (MAPE) were calculated to assess differences between each device and the criterion (MAPE <10% was considered as a threshold for validity).

**Results:**

In the field study of healthy individuals, all but one of the activity monitors showed a substantial correlation (CCC ≥0.95) with the criterion device and MAPE <10%. In patients with heart failure, the correlation of only two activity monitors (Garmin vívofit 3 and Withings Go) was classified as at least moderate (CCC ≥0.90) and none of the devices had MAPE <10%. In the lab-based study at speeds 4.2 and 3.6 km·h^-1^, all activity monitors showed substantial to almost perfect correlations (CCC ≥0.95) with the criterion and MAPE in the range 1%-3%. However, at slower speeds of 3.0 and 2.4 km·h^-1^, the accuracy of all devices substantially deteriorated: their correlation with the criterion decreased below 90% and their MAPE increased to 4–8% and 10–45%, respectively.

**Conclusions:**

Even though none of the tested activity monitors fall within arbitrary thresholds for validity, most of them perform reasonably well enough to be useful tools that clinicians can use to simply motivate chronic heart failure patients to walk more.

## Introduction

Increasing physical activity (PA) levels improves physical function and quality of life of patients with heart failure (HF), with both reduced and preserved ejection fraction [1–3]. Unfortunately, the levels of PA in patients with HF remain low and effective interventions to increase PA in this population are needed [4,5]. In healthy populations, consumer-level activity monitors (i.e. pedometers and accelerometers) are frequently used as motivational tools to increase PA [6–8] and many of them have been previously shown to be valid devices for assessing daily step count both in lab-based and free-living settings [9–11]. However, since those validation studies were conducted in healthy volunteers and didn’t examine the validity of the devices at slower walking speeds, it is not clear whether their results can be applied to patients with HF, who typically walk at slower speeds and shuffle their feet [12]. Considering how PA monitors must estimate step frequency based on accelerometry-based algorithms, feet shuffling, slower-than-normal walking speeds, and other characteristics can hamper the ability of activity monitors to accurately assess step count [13,14].

To date, only a few studies have evaluated the validity of consumer-level activity monitors for measuring step count in patients with HF and chronic conditions in general [15]. For example, a study in 24 patients with cardiac disease (10 of whom had HF) showed that to obtain an accurate measurement of steps using the Fitbit Zip, a speed of 3.6 km·h^-1^ or faster was required, which may be a challenge for patients with cardiac disease who normally walk at a slower pace [16]. Another study found that the Omron HJ-720ITC pedometer was accurate in persons with chronic obstructive pulmonary disease at walking speeds of 3.4 km·h^-1^ or faster, but it was less accurate at lower speeds [17]. Similarly, one of the few validation studies conducted in patients with HF demonstrated that the Omron HJ-720ITC pedometer, though accurate for monitoring steps in individuals with normal walking behavior, seems unsuitable for chronically ill patients characterized by slow walking gaits [18].

As a result of the rapidly evolving development of wearable electronics, many of the PA-tracking devices previously validated in patients with chronic conditions are not manufactured anymore. Furthermore, it is not clear whether the new models of the same manufacturer possess the same measurement characteristics as the older models (e.g. whether they use tri-axial or uni-axial acceleration data, or what they consider as the minimum threshold for detecting a step). Additionally, unlike many of the previously validated activity monitors that were often waist-worn [15–18], the new generation of consumer-level activity monitors are often designed in the form of a bracelet or smart watch to be worn on wrist. Though more convenient for most people, the ability of these wrist-worn devices to accurately count steps might be hampered by excessive arm movement as has been suggested by several studies [19,20]. Finally, most of the validation studies in chronically ill patients were conducted in lab-based settings [15,17,18] but their performance under free-living conditions is largely unknown.

To summarize, researchers and clinicians who are keen on implementing activity monitors during PA interventions for HF patients are inhibited in their desirable efforts due to the lack of validated activity monitors [15]. Thus, the objective of our study was twofold: (1) to evaluate the free-living validity of consumer-level activity monitors as measures of step count in patients with HF, and (2) to explore the accuracy of these devices in lab-based setting across a broad range of walking speeds, including slow walking speeds that are typical of HF patients.

## Methods

We evaluated six consumer-level activity monitors in a 3-day field study under free-living conditions and in a lab-based study at various speeds on a treadmill. The studies were reviewed and approved by the ethics committee of the Faculty of Physical Education and Sports, Charles University (198/2016), and they were conducted according to the principles of the Declaration of Helsinki. Participants were informed about all relevant aspects of the studies before enrolling, were notified about the right to refuse to participate or to withdraw consent at any time without reprisal, and then provided written informed consent.

### Consumer-level activity monitors

Six activity monitors were evaluated: Withings Go (WGO; Withings SA, Issy les Moulineaux, France), Fitbit Charge 2 (FC2; Fitbit, Inc., San Francisco, CA, USA), Garmin vívofit (GV1; Garmin Ltd., Schaffhausen, Switzerland) and Garmin vívofit 3 (GV3; Garmin Ltd., Schaffhausen, Switzerland), Omron HJ-322U-E (OMR; Omron Healthcare Co., Ltd., Kyoto, Japan), and SmartLAB walk+ (SLW; HMM Diagnostics GmbH, Dossenheim, Germany). Although there are numerous activity trackers on the market, we preferentially chose devices with replaceable button cell batteries that last for at least 6 months without needing to charge them, as we believed that for chronically ill (and often older) people, regular charging is an unnecessary burden that might decrease their adherence to a potential intervention [21]. The only exception to the no-charge rule was the FC2, which needs to be charged at least every 5th day. We included this device because the Fitbit company does not manufacture any wrist-worn model with replaceable button cell batteries, but we still wanted to include one of the Fitbit devices as they are among the most popular consumer-level activity monitors for use in research and in practice [22]. We also included two generations of the Garmin vívofit model (GV1 and GV3) to see if their characteristics are comparable. While most of the devices in our study are wrist-worn, we have also included the waist-worn OMR and the neck-worn SLW.

The WGO was worn as a wrist band on the non-dominant hand as recommended by the manufacturer. The FC2 can be worn on either the dominant or non-dominant hand. For the purpose of our study, it was worn on the wrist of the dominant hand. The GV1 and GV3 are two generations of the same wrist-worn model, and the manufacturer does not specify whether it needs to be worn on the dominant or non-dominant hand. To avoid bias resulting from overestimation when worn on a dominant hand, we choose to randomly alternate their placement between both arms, even though this solution introduced inter-wrist random errors. The GV1 and GV3 were always placed proximal to the WGO and FC2 devices. The OMR was worn attached to a belt at the left axillary line. The SLW was worn around the neck, tucked under the shirt, with the device dangling around the xiphoid process.

### Field study

To explore the validity of activity monitors under free-living conditions, a 3-day field study was conducted. In this study, both criterion validity, with step count recorded by Actigraph wGT3X-BT as the criterion, and concurrent validity were examined.

#### Study sample

For the field study, we recruited a convenience sample of 15 patients with HF and 14 healthy individuals, which is comparable to the sample size used in previous validation studies of PA monitors [10,15,23].

The HF patients were outpatients recruited from the General University Hospital in Prague (Czech Republic). The eligibility criteria included the diagnosis of HF according to the 2016 ESC Guidelines [24] with New York Heart Association (NYHA) class II or III. Patients with reduced (<40%), mid-range (40–49%), and preserved (≥50%) left ventricular ejection fraction were included. Patients were ineligible if they had symptoms of decompensated HF, recent (<3 months) myocardial infarction, or co-morbid conditions that would affect adherence to study procedures.

The healthy individuals, recruited via word of mouth, were ineligible if they self-reported suffering from HF, coronary heart disease, hypertension, stroke/transient ischemic attack, atrial fibrillation, type 2 diabetes, chronic obstructive pulmonary disease, painful conditions, depression, musculoskeletal or neurological disorders, or any other condition that might affect their physical fitness, gait characteristics, ambulatory behavior, or adherence to study procedures. Pregnant women were also excluded.

General characteristics (age, sex, handedness) and anthropometric measures (stature, body mass) were collected from all participants. For HF patients, NYHA class was assigned by the treating physician, and left ventricular ejection fraction (LVEF) was assessed by echocardiography performed within the three months preceding this study. For healthy individuals, LVEF was not assessed, as all of them were without chronic conditions and relatively fit; therefore, their LVEF can be expected to be within normal range for their age and sex.

For anthropometric measurements, the participants were asked to remove any footwear and to wear only light clothing. Stature was measured using a stadiometer to the nearest cm. Body mass was measured on a calibrated electronic scale to the nearest kg. Body mass index was calculated by dividing body mass (kg) by the square of the stature (m2). The sample characteristics of both groups are summarized in Table 1.

**Table 1:** Characteristics of the participants from the field study: Means (SD) or percentages.

	HF patients (n = 15)	Healthy individuals (n = 14)	p-value for a comparison between HF patients and healthy individuals
Age (yr)	65.5 (12.6)	43.3 (18.9)	0.42
Males (n)	9	5	0.35
Females (n)	6	9	0.35
Stature (cm)	172.3 (6.6)	169.4 (9.1)	0.47
Body mass (kg)	84.1 (15.7)	64.2 (10.4)	0.73
Body mass index (kg/m^2^)	28.2 (3.9)	22.3 (2.9)	0.78
Handedness (% of right-handed participants)	93	100	>0.99
NYHA class (% of patients with class II)	60	NA	NA
Heart failure with reduced ejection fraction (% of patients)	47	NA	NA
Ejection fraction (%)	39.2 (16.5)	NA	NA

#### Validation criterion

The accelerometer Actigraph wGT3X-BT (ActiGraph, Pensacola, FL, USA) was used as the criterion as it is a reliable and valid tool [25,26] that has been widely used in various populations, including low-activity patients with chronic conditions such as HF [7,15,27]. The device was worn at the waist on the right side, using the elastic belt provided by the manufacturer, and was positioned in line with the armpit and knee with the USB port cover facing up. The device was operated according to the manufacturer’s default settings (i.e. sampling rate of 30 Hz). ActiLife 6 (v6.13.3) software (ActiGraph, Pensacola, FL, USA) was later used to reintegrate data to 60-second epochs and calculate daily step count.

#### Procedures

The six consumer-level activity monitors and the Actigraph wGT3X-BT were fitted to the participants: FC2 on the dominant hand, WGO on the non-dominant hand, GV1 and GV3 randomly alternating between both hands (always proximal to FC2 and WGO), SLW around the neck, and OMR at the waist. The participants were first asked to remove and re-attach the devices to familiarize themselves with the routine under the supervision of the researchers and to prove that they are capable of adhering to the protocol. They were then instructed to wear all the devices simultaneously during waking hours, except during bathing and water-based activities, and return them 3 days later. In addition, they were asked to record when they removed the devices in a diary. Upon returning the devices, we discussed the diary records with the participants and asked them specifically about periods when the devices were not worn simultaneously. Subsequently, total daily step counts were recorded either directly from the display of the devices (OMR, SLW), or from the corresponding application after syncing (WGO, FC2, GV1, GV3). All days, at which participants wore the devices simultaneously, were included in the analyses, regardless of the total daily wear time.

### Lab-based study

To evaluate the validity of activity monitors during slow walking speeds, a lab-based study was conducted that included treadmill walking at various speeds. In this study, the criterion validity was assessed with manually counted steps as the criterion.

#### Study sample

For the lab-based study, we recruited a convenience sample of 20 healthy participants via word of mouth. The eligibility criteria were the same as for the healthy individuals in the field study and the participants’ characteristics are summarized in Table 2. We chose to recruit healthy participants rather than HF patients for the lab-based study, as we aimed to validate the devices across the broad spectrum of walking speeds (range 2.4 to 4.2 km·h^-1^) and we were not sure if the HF patients would have been able to safely achieve the speeds at the faster end of the spectrum.

**Table 2:** Characteristics of the participants from the lab-based study: Means (SD) or percentages.

	Healthy individuals (n = 20)
Age (yr)	34.3 (11.6)
Males (n)	15
Females (n)	5
Stature (cm)	177.8 (9.4)
Body mass (kg)	73.5 (10.8)
Body mass index (kg/m^2^)	23.2 (2.2)
Handedness (% of right-handed participants)	90

#### Validation criterion

In the lab-based study, manually counted steps were used as the criterion. The number of steps was counted independently by two researches (TV and JM) using a hand-tally counter. In the rare occasion when their observations were not in agreement (the difference was never greater than one step), the greater of the two values was recorded.

#### Procedures

Lab-based testing included treadmill walking at various speeds. All six consumer-level accelerometers were fitted to the participants as in the field study (in the lab-based study, the Actigraph wGT3X-BT was not employed, as the manually counted steps were used as the criterion). First, the participants were given 5 minutes to warm-up and familiarize with treadmill walking. Then, they walked for 3 min at speeds of 2.4, 3.0, 3.6, and 4.2 km·h^-1^ at 0% grade. The 3-min stages were chosen to be comparable with other studies [9,16,28]. The range of speeds was chosen so that the slowest speed corresponded to a self-selected walking speed in HF patients [29], and the fastest speed corresponded to the lower limit of moderate intensity PA [30]. Between each stage, the participants stood still with their hands on the handrails of the treadmill for 2 min while step counts were recorded from the display of the accelerometers.

### Data analysis

For the criterion validity, the concordance correlation coefficients (CCC) accompanied with the lower and upper bounds of the 95% confidence interval were used to evaluate the agreement between the consumer-level devices and the criterion by measuring the variation from the 45° line through the origin (the concordance line) [31]. To assess the degree of agreement, the calculated CCC was compared to the following strength-of-agreement criteria: <0.90 poor, 0.90 to 0.95 moderate, 0.95 to 0.99 substantial, >0.99 almost perfect [32]. Additionally, a hypothesis that the true regression line between the device and the criterion equals the concordance line was tested using post-hoc tests in a linear regression model with p-values being adjusted for multiple comparisons using the Holm’s method. Furthermore, mean percentage errors (MPE) and mean absolute percentage errors (MAPE) for the number of steps were calculated to assess differences between each device and the criterion. The MPE value assesses the degree of overall overestimation or underestimation for each device against the criterion, while the MAPE value provides the most relevant and comparable indicator of individual error since it accounts for both overestimation and underestimation from each participant [33]. Finally, Bland-Altmann charts were constructed to evaluate the mean bias and the limits of agreement between each device and the criterion [34].

For the concurrent validity, one-way random effects intraclass correlation coefficients (ICC) and their 95% confidence intervals were calculated to evaluate the agreement between the devices. The calculated ICC was used as the basis to assess the degree of agreement using the following guideline: <0.50 poor, 0.50 to 0.75 moderate, 0.75 to 0.90 good, >0.90 excellent correlation [35]. Furthermore, a linear mixed-effects model accompanied by post-hoc tests was used to identify potential differences in mean daily step counts between devices.

Differences in participants’ characteristics (age, sex, anthropometric measures) between HF patients and healthy controls in the field test were compared using the Wilcoxon rank sum test for ordinal and continuous variables, and the Pearson’s Chi-squared test for categorical variables. All data were tested for normality using the Shapiro-Wilk test. All statistical analyses were performed using the statistical package R (version 3.3.3), and a p-value of ≤0.05 was considered as statistically significant.

## Results

### Field study

In the field study, the mean daily number of steps based on the step counts measured by the Actigraph during full days (i.e. excluding the first and last day of the testing period) was 8995 (SD 5769) steps for healthy individuals and 6298 (SD 3088) steps among patients with HF. The outcome measures of the criterion validity in the field study are outlined in Table 3 (healthy individuals) and Table 4 (patients with HF). Data for the WGO are not reported for healthy individuals as the device failed during the testing period and we only yielded data from 4 participants. The outcomes for the FC2 include measurements from 12 healthy individuals and 13 patients with HF and the outcomes for the SLW and OMR include measurement from 14 patients with HF. All other outcomes include measurements from all participants and all the participants had at least two full days of measurement included in the analysis.

**Table 3:** Outcome measures of the criterion validity in the field study of healthy individuals.

Activity monitor	Mean (SD) [daily steps]	Difference vs criterion (LoA) [daily steps]	CCC (95% CI)	MPE	MAPE
OMR	8480 (5955)	-322 (-2730 to 2090)	0.97 (0.92–0.99)	-4%	8%
SLW	8573 (6022)	-230 (-2790 to 2330)	0.97 (0.92–0.99)	-3%	8%
GV1	8562 (5426)	-240 (-3180 to 2700)	0.95 (0.86–0.98)	-2%	10%
GV3	8983 (5737)	181 (-1980 to 2340)	0.97 (0.92–0.99)	+2%	7%
FC2	10876 (6424)	1070 (-1960 to 4100)	0.91 (0.75–0.97)	+12%	12%

OMR: Omron HJ-322U-E; SLW: SmartLAB walk+; GV1: Garmin vívofit; GV3: Garmin vívofit 3; FC2: Fitbit Charge 2; SD: standard deviation; LoA: limits of agreement; CCC: concordance correlation coefficient; CI: confidence interval; MPE: mean percentage error; MAPE: mean absolute percentage error. MPE and MAPE values closer to 0 are desired.

**Table 4:** Outcome measures of the criterion validity in the field study of HF patients.

Activity monitor	Mean (SD) [daily steps]	Difference vs criterion (LoA) [daily steps]	CCC (95% CI)	MPE	MAPE
WGO	4516 (2987)	-204 (-2350 to 1140)	0.90 (0.77–0.96)	-15%	18%
OMR	4297 (2463)	-475 (-2370 to 1420)	0.82 (0.56–0.93)	-9%	12%
SLW	4299 (2483)	-474 (-2210 to 1260)	0.84 (0.65–0.93)	-7%	13%
GV1	5921 (3621)	801 (-1440 to 3040)	0.89 (0.75–0.96)	+16%	18%
GV3	5671 (3510)	551 (-1560 to 2660)	0.92 (0.78–0.97)	+10%	13%
FC2	6796 (4660)	2120 (-2360 to 6600)	0.48 (0.20–0.69)	+46%	46%

WGO: Withings Go; OMR: Omron HJ-322U-E; SLW: SmartLAB walk+; GV1: Garmin vívofit; GV3: Garmin vívofit 3; FC2: Fitbit Charge 2; SD: standard deviation; LoA: limits of agreement; CCC: concordance correlation coefficient; CI: confidence interval; MPE: mean percentage error; MAPE: mean absolute percentage error. MPE and MAPE values closer to 0 are desired.

In healthy individuals, all devices except one showed substantial correlation (CCC >0.95) with the criterion device and MAPE <10% which is considered as a threshold for validity of activity monitors [36]. The only exception was the FC2 where the correlation was only moderate and MAPE was 12%. In concurrent validation (including the Actigraph), the devices showed excellent correlation (ICC = 0.92, 95% CI 0.84–0.97) but the linear mixed-effects model including all devices revealed significant differences in mean daily step counts between devices (p<0.01). However, in the linear mixed-effects model, which included all but the FC2 devices, the differences became insignificant (p = 0.37).

**Fig 1:**
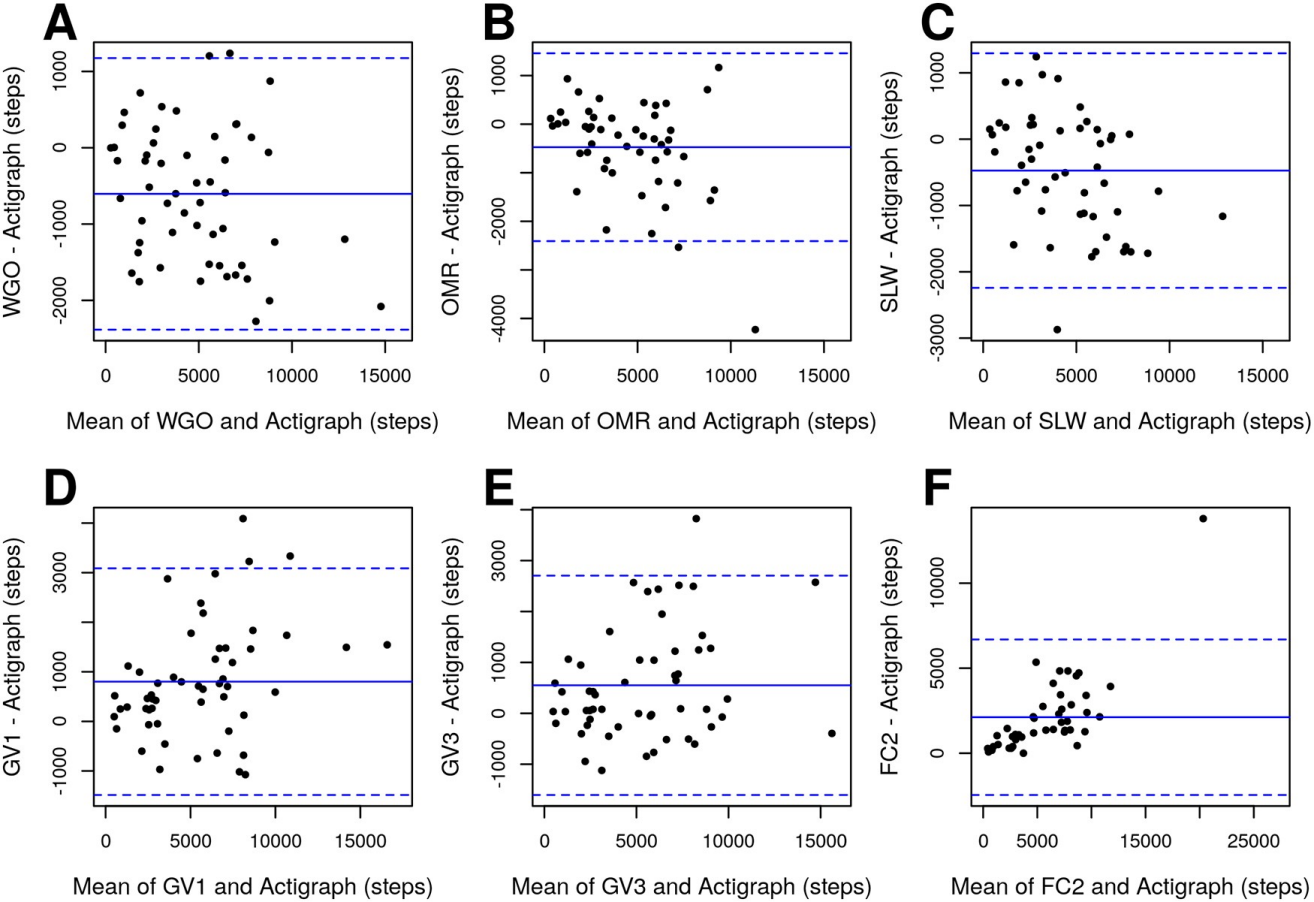
Bland-Altman plots for step count in patients with HF under free-living conditions. (A) Withings Go; (B) Omron HJ-322U-E; (C) SmartLAB walk+; (D) Garmin vívofit; (E) Garmin vívofit 3; (F) Fitbit Charge 2. The solid lines indicate the mean step count difference between the criterion device (Actigraph) and each of the evaluated consumer-level activity monitors. The dashed lines represent limits of agreement (±1.96*SD).

In patients with HF, the correlation of only two devices (GV3 and WGO) was classified as at least moderate (CCC >0.90), however all devices had CCC in the relatively narrow range from 0.82 to 0.92 and their MAPE was in the range of 12–18%, with the exception of the FC2 that showed poor correlation and high MAPE of 46%. The WGO, OMR, and SLW underestimated the number of steps by 7%, 9%, and 15%, respectively, while the GV3 and GV1 overestimated the number of steps by 10% and 16%, respectively. In concurrent validation, the devices showed only moderate correlation (ICC = 0.60, 95% CI 0.39–0.82) and the linear mixed-effects model revealed significant differences in mean daily step counts between devices. After excluding the FC2 from the analysis the correlation improved to good (ICC = 0.76, 95% CI 0.59–0.90) but the differences between devices remained significant. The Bland–Altman plots revealed no systematic differences between the activity monitors and Actigraph (Fig 1).

### Lab-based study

The outcome measures of the criterion validity in the lab-based study are shown in Table 5. Data for the WGO are not reported as the device failed during the testing period and only yielded measurements from six participants. One measurement with the GV3 at the speed of 4.2 km·h^-1^ was excluded from the analysis as it was an extreme outlier likely due to an error in reading or recording the value.

**Table 5:** Outcome measure of the criterion validity in the lab-based study at various treadmill speeds.

Activity monitor	Speed [km·h^-1^]	CCC (95% CI)	MPE	MAPE
OMR	4.2	0.99 (0.97–1.00)	-1%	1%
	3.6	0.99 (0.98–1.00)	-1%	1%
	3.0	0.21 (0.00–0.52)	-7%	7%
	2.4	0.11 (0.00–0.22)	-44%	45%
SLW	4.2	0.98 (0.95–0.99)	0%	1%
	3.6	0.99 (0.97–0.99)	0%	1%
	3.0	0.59 (0.24–0.81)	-3%	4%
	2.4	0.22 (0.00–0.41)	-18%	18%
GV1	4.2	0.96 (0.91–0.98)	-2%	2%
	3.6	0.95 (0.89–0.98)	-2%	3%
	3.0	0.66 (0.40–0.82)	-7%	7%
	2.4	0.11 (0.00–0.29)	-24%	24%
GV3	4.2	0.97 (0.92–0.99)	-1%	1%
	3.6	0.99 (0.97–1.00)	-1%	1%
	3.0	0.46 (0.10–0.72)	+4%	8%
	2.4	0.45 (0.20–0.65)	+4%	13%
FC2	4.2	0.90 (0.78–0.96)	-2%	2%
	3.6	0.99 (0.98–1.00)	-1%	1%
	3.0	0.82 (0.68–0.90)	0%	4%
	2.4	0.38 (0.00–0.67)	+3%	10%

OMR: Omron HJ-322U-E; SLW: SmartLAB walk+; GV1: Garmin vívofit; GV3: Garmin vívofit 3; FC2: Fitbit Charge 2; CCC: concordance correlation coefficient; CI: confidence interval; MPE: mean percentage error; MAPE: mean absolute percentage error. MPE and MAPE values closer to 0 are desired.

At speeds 3.6 and 4.2 km·h^-1^, all devices showed substantial to almost perfect correlation (CCC >0.95) with the criterion (with the exception of the FC2 at 4.2 km·h^-1^ where the correlation was only moderate) and MAPE in the range 1% - 3%. At slower speeds of 3.0 and 2.4 km·h^-1^, none of the devices showed a moderate (or better) correlation with the criterion and MAPE increased to 4–8% and 10–45%, respectively.

## Discussion

We evaluated the validity of several consumer-level activity monitors for measuring step count in patients with HF and in healthy individuals in lab-based and free-living settings. In the free-living setting, we found that all of the devices performed worse in patients with HF than in healthy individuals, with only two devices (GV3 and WGO) showing a moderate correlation with the criterion but none of the devices being within the 10% range for MAPE, which has been suggested as acceptable pedometer performance under free-living conditions. During the lab-based setting, we tested these devices during treadmill walking at several walking speeds (2.4–4.2 km·h^-1^) and showed that the accuracy of these devices decreases with slower treadmill speeds, which can partially explain their limited performance in patients with HF who walk slower than healthy individuals in free-living settings [29].

Although the purpose of this study was not to make a recommendation to purchase any of the tested activity trackers, the GV3 seems to be the most appropriate for use in slow walking populations like HF patients because it displayed the highest CCC (0.92) and reasonable MAPE (13%) along with consistent performance across the various speeds on the treadmill. On the opposite end of the spectrum was the FC2; despite being the most accurate at lower speeds during treadmill walking, it failed in free-living testing in HF patients with CCC of 0.48 and MAPE of 46%. Therefore, the FC2 is the only device that cannot be recommend for potential use in this population. As for the remaining devices, none of them proved consistently better or worse than the others. When comparing the two generations of the Garmin models, the outcomes of the older GV1 had a similar pattern as the newer GV3, but in most tests, the GV1 had slightly worse performance than the GV3.

As for the comparison between the wrist-worn and torso-worn PA trackers used in the present study, we did not observe any systematic differences. For example, the SLW scored the second best MAPE in free-living testing in HF patients and excelled at 3.0 km·h^-1^ during treadmill walking, but it was only average at 2.4 km·h^-1^ on the treadmill. Similarly, the OMR, despite having the best MAPE in the free-living setting in HF patients, was clearly the least accurate device when tested at 2.4 km·h^-1^ on the treadmill. Interestingly, at the slowest treadmill walking speed, the GV3 and FC2, both wrist-worn devices, achieved better MAPE values (13%, and 10%, respectively) than the waist-worn OMR (45%) and the neck-worn SLW (18%). A similar tendency was observed previously and may be explained by the fact that false steps elicited by arm movements of wrist-worn monitors compensate for non-detected steps during slow walking [37].

To summarize, all tested consumer-level activity monitors performed worse in HF patients than in healthy individuals which can be partially explained by the lower accuracy of these devices at slower speeds. Of the devices that we evaluated in our study, the GV3 seemed to perform best in HF patients, while the FC2 failed to detect steps accurately under free-living conditions in this population.

### Results in the context of other literature

Of the activity monitors validated in this study, the GV1 has the largest body of validation studies. In a lab-based study, An et al. [28] tested 10 activity monitors in healthy adults on a treadmill at several speeds starting at 3.2 km·h^-1^. For the GV1, the MAPE value for treadmill walking averaged 5.9% with no indication that the accuracy of the devices deteriorated at slower speeds. This finding is consistent with our study where the accuracy of the GV1 started to decrease from 3.0 km·h^-1^ and slower. Furthermore, Ehrler et al. [37] tested the GV1 at three slow walking speeds over ground (1.44, 2.16, and 2.88 km·h^-1^) with the resulting MAPE values of 80.1%, 12.2%, and 5.3%. These values demonstrate higher accuracy at slow walking speeds than our observations (MAPE 24% for the slowest speed of 2.4 km·h^-1^). However, the authors of that study imposed the slow walking speed using a combination of fixed step length (using a string between legs) and fixed cadence dictated by a metronome—a rather unnatural setting that cannot be directly compared to slow walking on a treadmill. Finally, Chen et al. [38] tested the GV1 during treadmill walking (starting from 3.2 km·h^-1^) and jogging with MAPE values between 1.5 and 4.2%, similar to what we observed at the corresponding speeds in our study. Therefore, our data generally agree with the data from other studies in that the GV1 is acceptable to use at normal walking speeds but becomes less acceptable as speed progressively decreases below 3.0 km·h^-1^.

In addition to lab-based testing, several studies validated the GV1 under free-living conditions. A study comparing the GV1 in healthy adults against the same model of Actigraph as in our study found that MAPE was 12.5%, which is only slightly higher than in our healthy adults (10%) and lower than in the HF patients (18%) [11]. Other studies found higher values of MAPE in healthy adults under free-living conditions (17.8% and 22.9%) but these studies did not use the Actigraph as a criterion (the New Lifestyle NL-1000 Series and Yamax SW-200 were used in these studies as the criterion), thus we cannot directly compare them with the results of our study [28,39].

Apart from the GV1, the reports of the validity of other devices are scarce. In one free-living study, the FC2 overestimated steps by 32.2% compared to the Actigraph [40]. This value is between the corresponding values found in our study for healthy individuals (12%) and HF patients (46%). Additionally, the OMR was tested in a structured lab-based protocol combining treadmill walking at various speeds, treadmill running (9.66 km·h^-1^), over-ground walking under various conditions (hands in pocket, pushing a stroller, etc.), and several activities of daily living. During this combined protocol, the OMR was found to underestimate the number of steps by 8.4% with a MAPE of 14.2% [41]. Though not directly comparable with our protocol, these values are consistent with our observation. Finally, the validity of the GV3 together with the GV1 was explored in adolescents in free-living settings with the Yamax Digiwalker SW-701 as the criterion and the resulting MAPE values were 11.5% and 11.8, respectively [42].

In the current body of literature, little evidence supports the validity of the devices evaluated in our study in general population, with the exception of the GV1. Also, no study had examined their accuracy in patients with chronic conditions such as HF. Although the purpose of this study was not to compare the walking characteristics of healthy individuals to HF patients, healthy individuals were used to compare the findings of our study to other studies. As a result, our findings from the lab-based study and free-living conditions in healthy individuals agree with those from other studies. Therefore, we can assume that our data accurately reflect the true performance of these six devices in HF patients as well.

### Study limitations

To the best of our knowledge, this is the first study that validated consumer-level activity monitors in HF patients during a free-living setting and its results can help both researchers and practitioners choose the right activity monitor for their HF patients. However, this study is not without limitations.

First, we used the Actigraph as the step-counting criterion for the field study as it is well-validated in patients with chronic diseases, and it has been widely used in other validation studies as a criterion. However, the Actigraph counts steps based on accelerometry and thus may suffer from inaccuracies at lower speeds, not unlike the consumer-level activity monitors that were used in the present study. Indeed, when tested during a 2-minute walk test in adults aged over 60, the Actigraph appeared to underestimate step count compared to visually counted steps [43]. Other studies showed that the number of steps detected by Actigraphs can be significantly different from manually-counted steps [44], especially at very slow speeds [20].

Second, the mean daily step count of HF patients in our study was 6298 which is relatively high for HF patients [45,46]. Thus, we cannot be sure that our results apply to all HF patients, especially those who walk less and with possibly slower paces. Therefore, future research should seek to employ similar study designs as the one used in the present study but using various HF populations.

## Conclusions

Our study demonstrated that consumer-level activity monitors are accurate at measuring steps in healthy individuals during normal walking speeds (≥ 3.6 km·h^-1^) but their accuracy decreases in patients with HF, especially with lower walking speeds (≤ 3.0 km·h^-1^).

Even though none of the tested activity monitors fall within arbitrary thresholds for validity, most of them perform reasonably well enough to be useful tools for clinicians to motivate patients with HF to walk more. Specifically, of the devices tested in the present study, we can recommend the GV3 as it demonstrated the highest correlation with the criterion device and the lowest MAPE both in HF patients and at slow walking speeds.

In research settings, however, the use of consumer-level devices in slower walking populations needs to be carefully weighed, and their potential inaccuracy needs to be taken into account when interpreting their outcomes. Given that even research-grade devices appear to be inaccurate in measuring steps in slow-walking populations, future research is required to explore alternative placements of activity monitors (e.g. ankle [20]) as a way to achieve more accurate measurements in patients with HF.

## Supporting information

S1 Appendix Data tables. (XLSX)
